# Urgent-start peritoneal dialysis for patients with end stage renal disease: a 10-year retrospective study

**DOI:** 10.1186/s12882-019-1408-9

**Published:** 2019-07-02

**Authors:** Hongjian Ye, Xiao Yang, Chunyan Yi, Qunying Guo, Yafang Li, Qiongqiong Yang, Wei Chen, Haiping Mao, Jianbo Li, Yagui Qiu, Xunhua Zheng, Dihua Zhang, Jianxiong Lin, Zhijian Li, Zongpei Jiang, Fengxian Huang, Xueqing Yu

**Affiliations:** 1 Department of Nephrology, The First Affiliated Hospital, Sun Yat-sen University, 58th, Zhongshan Road II, Guangzhou, 510080 China; 2 Key Laboratory of Nephrology, Ministry of Health and Guangdong Province, Guangzhou, 510080 Guangdong China

**Keywords:** End stage renal disease, Peritoneal dialysis, Urgent-start peritoneal dialysis, Technique survival, Catheter patency, Complications

## Abstract

**Background:**

Urgent-start peritoneal dialysis (PD) can help patients with end-stage renal diseases (ESRD) that are referred late to dialysis. However, catheter patency and related complications of urgent-start PD have not been thoroughly clarified. We investigated the clinical outcomes of urgent-start PD in a Chinese cohort.

**Methods:**

We enrolled ESRD patients who received urgent-start PD (starting PD within 14 days after catheter insertion) in our center from January 1, 2006 to December 31, 2014, and followed them up for 10 years. The primary outcome was catheter failure. Secondary outcomes included short-term and long-term complications related to urgent-start PD.

**Results:**

Totally 2059 patients (58.9% male, mean age 47.6 ± 15.9 years) were enrolled. Few perioperative complications were observed, including significant hemorrhage (*n* = 3, 0.1%) and bowel perforation (*n* = 0). Early peritonitis occurred in 24 (1.2%) patients (0.28 episodes per patient-year). Within the first month after catheter insertion, functional catheter malfunction occurred in 85 (4.1%) patients, and abdominal wall complications (including hernia, hydrothorax, hydrocele, and leakage) in 36 (1.7%) patients. During a median 36.5 (17.7–61.4) months of follow-up, 75 (3.6%) patients experienced catheter failure, and 291 (14.1%) had death-censoring technique failure. At the end of 1-month, 1 -year, 3-year, and 5-year, catheter patency rate was 97.6, 96.4, 96.2, 96.2%; and technique survival rate was 99.5, 97.0, 90.3, 82.7%, respectively. After adjusting for confounders, every 5-year increase in age was associated with 19% decrease of risk for catheter failure (hazard ratio [HR]: 0.81, 95% confidence interval [CI]: 0.73–0.89). Male sex (HR: 1.43, 95% CI: 1.00–2.04), diabetic nephropathy (HR: 1.56, 95% CI: 1.08–2.25) and low hemoglobin levels (HR: 0.89, 95% CI: 0.81–0.98) were independent risk factors for abdominal wall complications.

**Conclusions:**

Urgent-start PD is a safe and efficacious option for unplanned ESRD patients. A well-trained PD team, a standardized catheter insertion procedure by experienced nephrologists, and a carefully designed initial PD prescription as well as comprehensive follow-up care, might be essential for the successful urgent-start PD program.

**Electronic supplementary material:**

The online version of this article (10.1186/s12882-019-1408-9) contains supplementary material, which is available to authorized users.

## Background

End stage renal disease (ESRD) has become a heavy burden to public health worldwide. According to the 2017 United States Renal Data System (USRDS) report, the crude incidence of ESRD diagnoses was 378 per million/year. Suprisingly, even with regular nephrology follow-up, more than 60% of the new diagnosed ESRD patients do not have clear plans when they begin renal replacement therapy, which prodispose them to a need for urgent dialysis [1]. China is limited in some healthcare resources, particularly in rural and remote areas [2]. A cross-sectional survey in China showed the overall prevalence of chronic kidney disease (CKD) to be 10.8%, with wide regional variations [3]. As most CKD patients are referred late to dialysis, unplanned dialysis is common in China [4, 5]. Moreover, as hemodialysis (HD) is unavailable in most rural or remote areas in China, peritoneal dialysis (PD) is the only option [6].

As a home-based renal replacement modality, PD has been increasingly used by ESRD patients. By the end of 2016, China had 74,138 registered patients on PD [7]. Sterile surgical technique is the most frequently used method for PD catheter placement in our region. Previous guidelines recommended that catheter insertion should be performed at least 2 weeks before starting PD [8, 9]. However, it is unrealistic for these unplanned dialysis patients to wait for a two-week’s period before initiating PD therapy. Urgent-start PD has been suggested to be an effective approach to prompt initiation of PD after catheter insertion, may avoid increased risk of central venous catheter-related complications, including bacteremia, central venous stenosis, and thrombosis associated with temporary use of HD [5, 10]. However, the possibility of adverse effects from urgent-start PD, such as catheter dysfunction, fluid leakage, and early infection, was a major impediment to its wider use [8, 11]. Although several studies have reported complications related to urgent-start PD, their evidence was relatively weak due to limited sample size and regional differences [5, 11–16].

In our center, almost all patients with ESRD experienced urgent catheter insertion and immediate initiation of PD therapy. We therefore investigated the prevalence of complications and outcomes of urgent-start PD for these patients.

## Methods

### Study population

We retrospectively enrolled all patients treated at our hospital from January 1, 2006 to December 31, 2014. All patients satisfied the following inclusion criteria: (1) diagnosed with ESRD; (2) Tenckhoff catheters were inserted with sterile surgical technique by nephrologists; and (3) initiated PD therapy within 14 days after catheter insertion.

### Urgent-start PD program

**Fig. 1:**
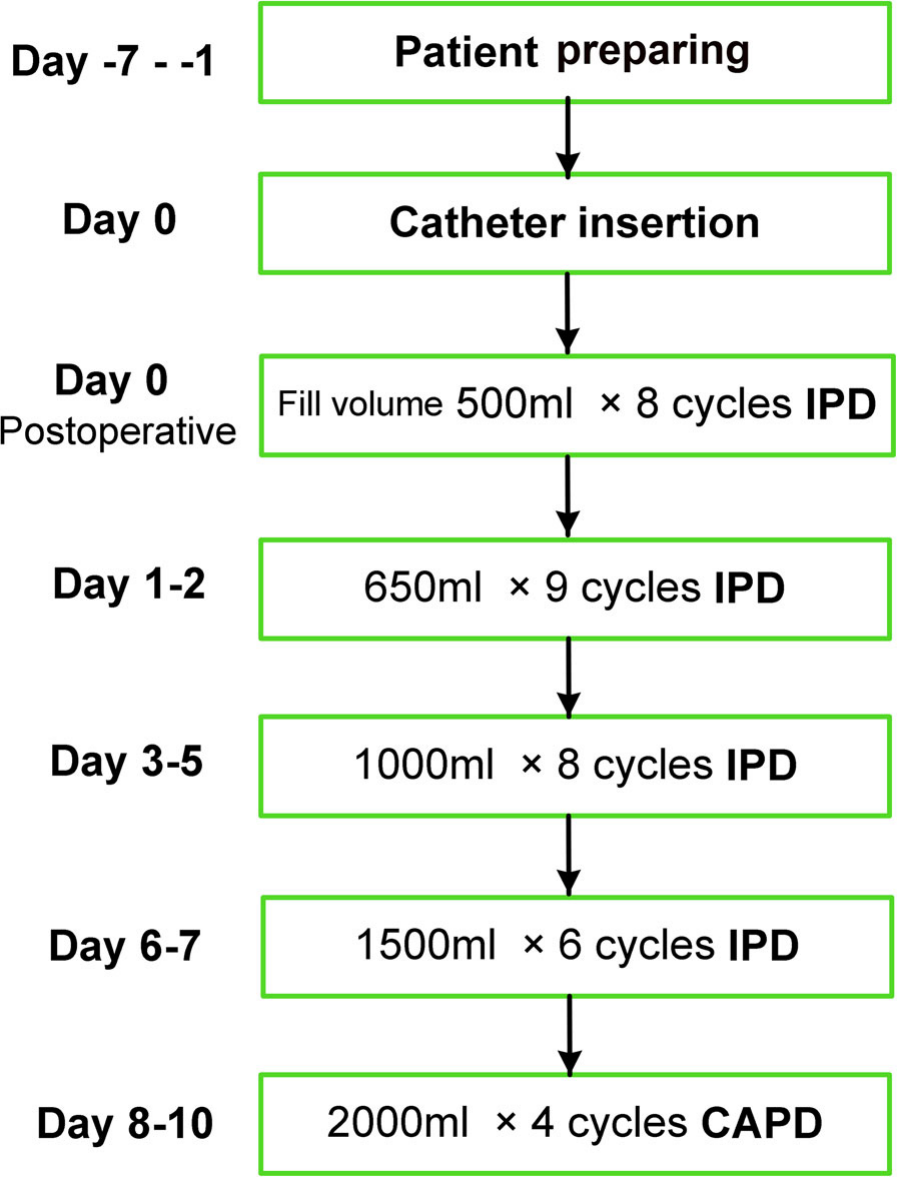
The flow chart of the urgent-start PD program

**Fig. 2:**
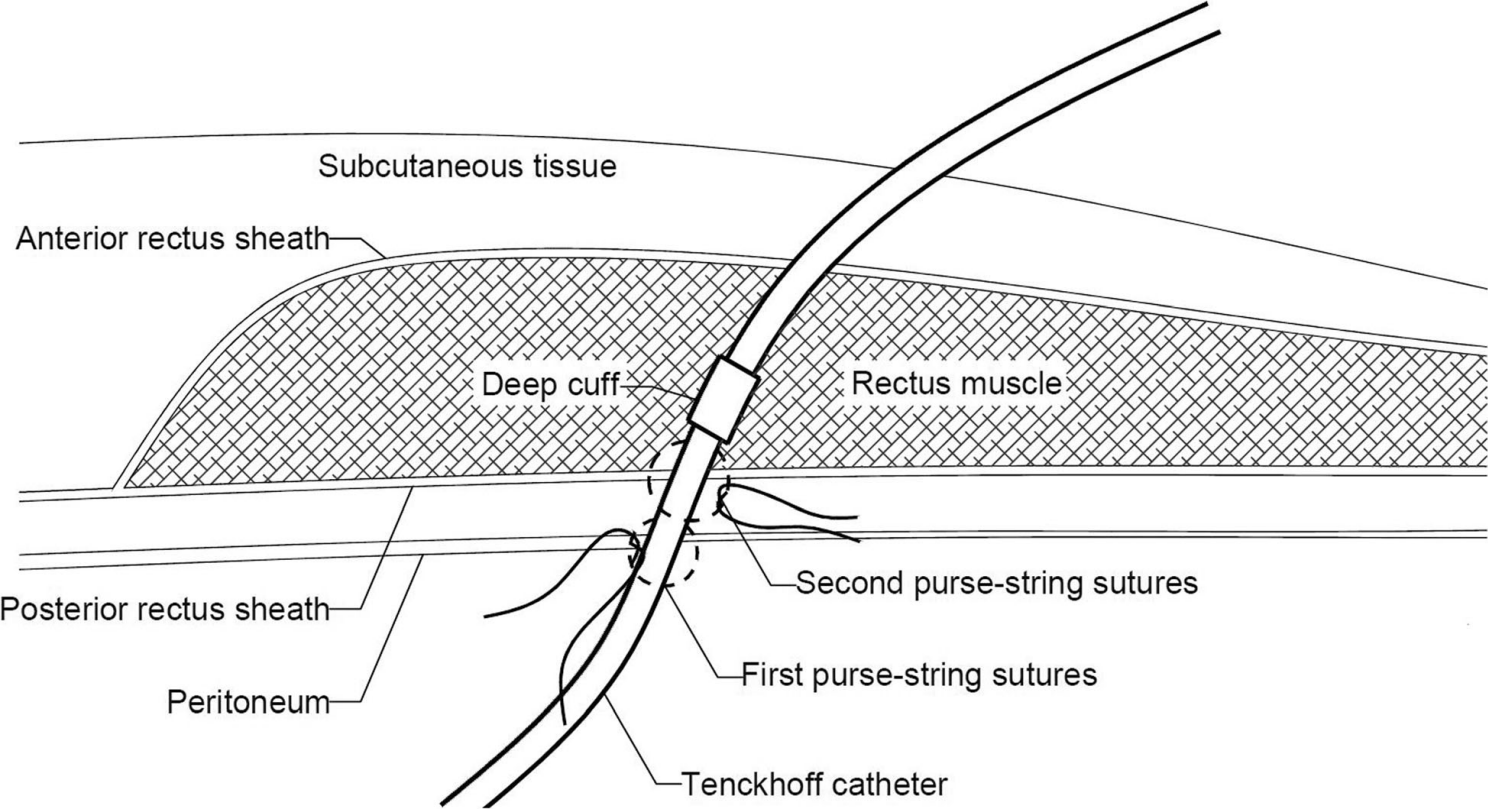
A schematic diagram of catheterization. A double purse-string sutures technique was used for the catheter fixation and prevention of leakage

Figure 1 shows the flow chart of the urgent-start PD program, which was defined as starting PD within 14 days after catheter insertion [16]. ESRD Patients who chose PD were placed double-cuff Tenckhoff catheters with open laparotomy technique by experienced nephrologists. A double purse-string suture technique on the posterior rectus sheath and parietal peritoneum was used for catheter fixation and prevention of leakage (Fig. 2). The deep cuff was placed into the rectus abdominis muscle. Intravenous cefuroxime or cefazolin was used as a prophylactic antibiotic 30 min before catheterization, unless the patient was allergic to penicillin. For those allergic to penicillin, we used single-dose ciprofloxacin for antibiotic prophylaxis. Intermittent peritoneal dialysis (IPD) treatment was initiated immediately after catheter insertion with a dialysate volume of 500 ml per dwell for 1 h (8 cycles per day) in first day at the supine position, then 650 ml per dwell for 1 h (9 cycles per day) in the next 1–2 days, and gradually increased to 2000 ml or the maximum tolerable volume within 8 to 10 days. According to the program, the IPD regime took about 6–9 h each day. Patients then began continuous ambulatory peritoneal dialysis (CAPD) (Fig. 1). A professional nursing team managed the IPD dialysate exchanges. Patients and their caregivers received a standard training program within 5–7 days after catheterization. Post-discharged PD patients were managed according to our follow-up program [17].

### Baseline demographic and laboratory data, and study outcomes

Demographic and clinical data, including age, sex, causes of renal diseases, comorbidity conditions, history of abdominal surgery, body mass index (BMI), and body surface area (BSA) were collected before catheterization. Laboratory data were also evaluated at the baseline. Residual renal function at the initiation of PD was estimated by the CKD-EPI equation [18]. We followed up the patients until any cause of dropping out of PD (death, change to HD, kidney transplantation, loss to follow-up) or December 31, 2016.

The primary outcome was catheter failure, which was defined as functional catheter problems that required catheter manipulation or replacement, or lead to technique failure. Catheter patency was defined as continuation of PD without any catheter manipulation or surgical intervention [9]. Secondary outcomes included death-censoring technique failure, functional catheter problems, and other urgent-start PD-related complications. Death-censoring technique failure was defined as transferring to HD from any cause; death, kidney transplantation, loss to follow up, transfer to other centers, and recovery of renal function were considered as censoring events [19]. Functional catheter problems included any troublesome inflow of dialysate and/or outflow of dialysate that possibly needed surgical intervention [20]. Omental wrap was diagnosed by opening procedures in this study. Short-term and long-term urgent-start PD-related complications, such as perioperative complications (including significant hemorrhage requiring transfusion or surgical intervention, bowel perforation), early infections (exit-site infections and peritonitis within 2 weeks after catheter insertion), and abdominal wall complications (including pericatheter leakage, subcutaneous leakage, hernia, hydrothorax, and hydrocele) [9], were recorded during the follow-up period. A PD team of three nephrologists reviewed individual medical records and identified causes for catheter failure, functional catheter problems, or technique failure.

### Management of functional catheter problems and abdominal wall complications

**Fig. 3:**
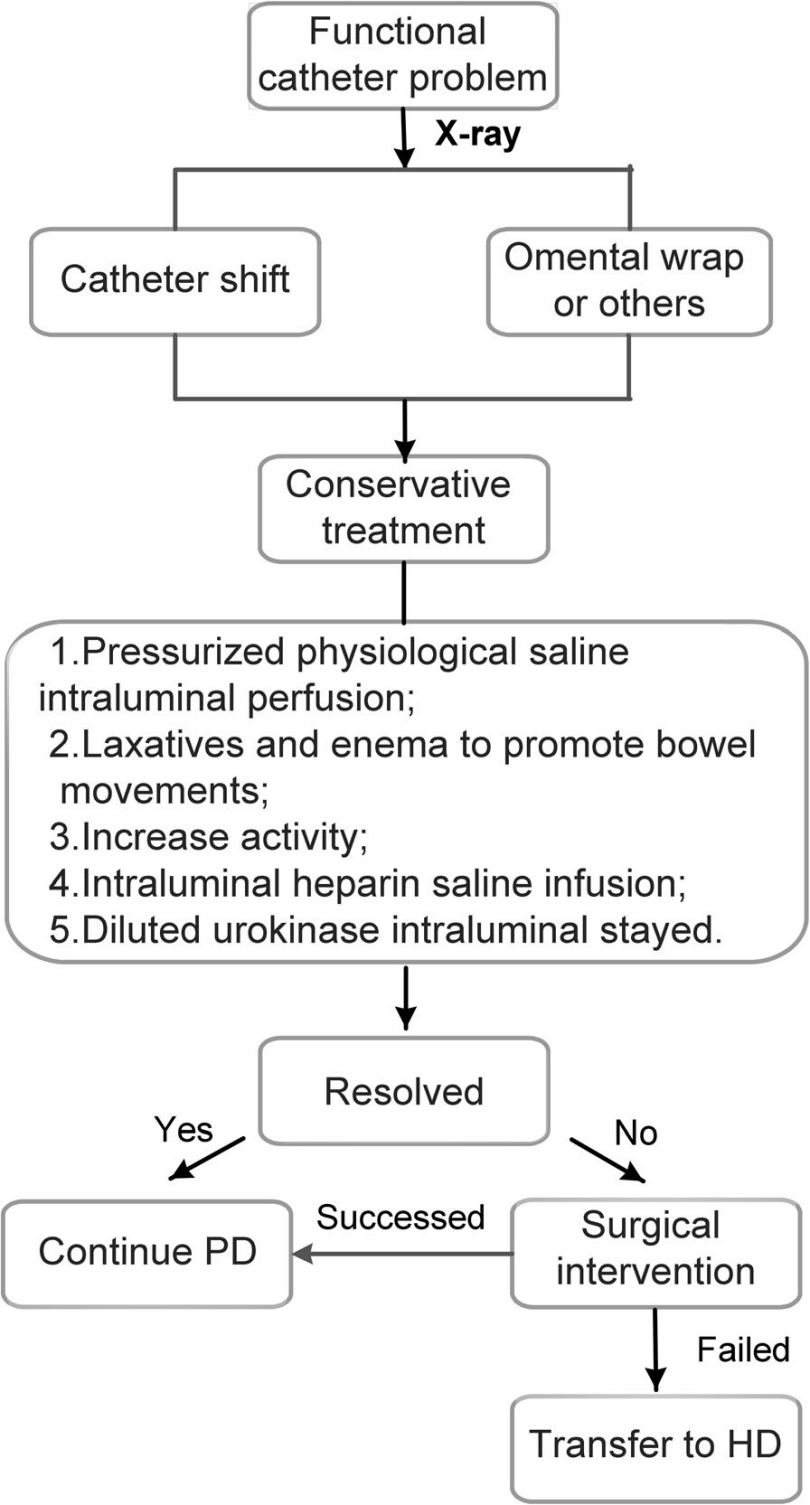
The flow chart of the management of functional catheter problems

Generally, a patient with functional catheter problem would receive an abdominal X-ray examination to identify whether the catheter had shifted. The drainage bag with dialysate was also examined to find any underlying cause for catheter obstruction. All these patients would initially receive conservative treatments. If the problem was unresolved, surgical interventions such as catheter manipulation, revision, or replacement, were conducted in the case of patient informed consent (Fig. 3).

Patients with abdominal wall complications would receive decreased infused volume, or temporarily suspended PD, or temporary HD therapy to allow for the possibility of natural repair (such as scrotal edema, pericatheter leakage, and subcutaneous leakage). If the problems were unresolved, the patients would receive surgical manipulation or transfer to permanent HD, as appropriate.

### Statistical analysis

We expressed the results as mean ± standard deviation (SD), frequencies (percentages),, and median (interquartile range [IQR]) according to the types of variables. The independent sample *t*-test was used for normally distributed continuous variables. The Mann–Whitney U-test was used to compare non-normal distributed continuous variables. For categorical variables, the Chi-square test was used. We apply Kaplan–Meier analysis to evaluate the catheter survival and technique survival. The Cox proportional hazards regression models were used to explore risk factors associated with catheter failure and abdominal wall complications. Data were analyzed with SPSS software (version 19.0). *P* < 0.05 was considered statistically significant.

## Results

### Patient characteristics

We included 2059 PD patients who met the inclusion criteria at our center: almost all patients initiated PD therapy immediately after catheter insertion and only a few patients who had weakness of peritoneum and rectus abdominis sheath would delay several days (within 14 days after catheter insertion) to start dialysis. Their mean age was 47.6 ± 15.9 years; 1212 (58.9%) patients were male. The most common cause of ESRD was chronic glomerulonephritis (58.8%), followed by diabetic nephropathy (21.8%) (Table 1). All the patients were inserted with Tenckhoff catheters using sterile open surgery by experienced nephrologists. Coiled catheters only accounted for 7.4%, the rest were straight catheters. Generally, patients started IPD treatment immediately after catheter insertion with infused dialysis volume gradually increasing to 2000 ml or the maximum tolerable volume, and then started on the regular CAPD regimen as described in Fig. 1.

**Table 1:** Demographic characteristics and clinical data

Variables	Patients (*n* = 2059)	Catheter failure (*n* = 75)	Catheter patency (*n* = 1984)	*P* value
Age (years)	47.6 ± 15.9	37.9 ± 15.0	47.9 ± 15.8	< 0.001
Sex (Male, n, %)	1212 (58.9%)	45 (60%)	1167 (58.8%)	0.905
BMI (kg/m^2^)	21.5 ± 3.2	20.7 ± 3.1	21.6 ± 3.2	0.020
BSA (m^2^)	1.59 ± 0.17	1.56 ± 0.15	1.58 ± 0.17	0.237
Primary renal disease (n, %)				0.905
Glomerulonephritis	1204 (58.5%)	46 (61.3%)	1158 (58.4%)	–
Diabetic nephropathy	449 (21.8%)	14 (18.7%)	435 (21.9%)	–
Hypertensive nephropathy	151 (7.3%)	5 (6.7%)	146 (7.4%)	–
Others	255 (12.4)	10 (13.3%)	245 (12.3%)	–
Diabetic nephropathy (n, %)	449 (21.8%)	14 (18.7%)	435 (21.9%)	0.502
Type of catheter (coiled VS. straingt)(n, %)	152 (7.4%)	11 (14.7%)	141 (7.1%)	0.014
History of abdominal surgery (n, %)	149 (7.2%)	8 (10.7%)	141 (7.1%)	0.252
Serum creatinine (mg/dL)	9.7 (7.7–12.2)	9.7 (7.7–12.1)	10.7 (8.4–13.7)	0.024
eGFR (ml/min/1.73m^2^)	5.0 (3.9–6.6)	5.0 (3.9–6.6)	4.7 (3.5–6.8)	0.384
Hemoglobin (g/dL)	8.1 ± 1.9	7.8 ± 1.5	8.1 ± 1.9	0.138
Serum albumin (g/dL)	3.5 ± 0.5	3.5 ± 0.6	3.5 ± 0.5	0.929

NOTE. Values expressed as mean ± SD, median (interquartile range), or number (percent); Abbreviations: *BMI* body mass index, *BSA* body surface area, *eGFR* estimated glomerular filtration rate. SI units conversions: serum creatinine, 1 mg/dL = 88.4 μmol/L; hemoglobin, g/dL = 1/10 g/L; serum albumin, g/dL = 1/10 g/L.

### The prevalence of functional catheter problems and catheter failure

**Fig. 4:**
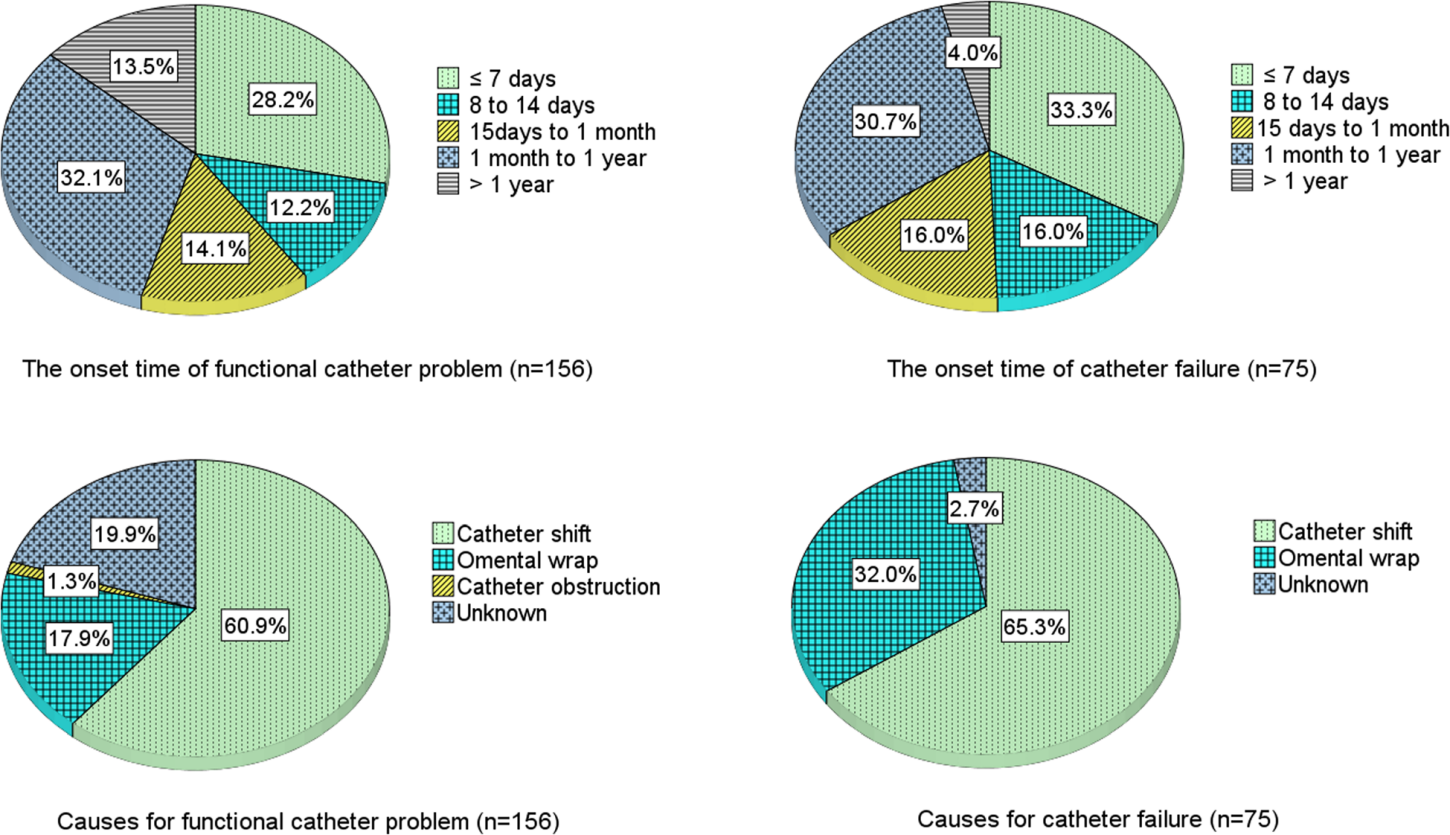
The distributions of the onset time and causes of the functional catheter problems and catheter failure

During a median of 36.5 months (IQR: 17.7–61.4 months) of follow-up, 156 (7.6%) patients experienced functional catheter problems. Among them, 28.2% events occurred within 7 days after catheter insertion, 12.2% occurred between 8 and 14 days, 14.1% occurred between 15 days and 1 month, and 32.1% occurred between 1 month and 1 year. The distribution of onset time for catheter failure was similar (Fig. 4**)**. According to clinical and intraoperative assessments, 60.9% of the functional catheter problems were caused by catheter shift, 17.9% caused by omental wrap, and 19.9% from unknown causes. Similarly, catheter shift (65.2%) was the leading cause for catheter failure; whereas omental wrap accounted for a higher proportion of causes for catheter failure at 32.0%, and unknown causes were only 2.7%.

All 156 patients firstly received series of conservative treatments. Among them, 81 (51.9%) catheters were rescued and 75 (48.1%) patients had catheter failure. Catheter survival rate was 97.6, 96.4, 96.2, 96.2, and 96.2%, respectively at the end of 1-month, 1-year, 3-year, 5-year, and 10-year. The successful rescue rate for catheters was 41.3% for events occurring within 14 days after insertion, 54% for events occurring between 1 month and 1 year, and 85.7% for events occurring after the first year; and was highest in patients with catheter obstruction (100%) or unknown causes (93.5%), 48.4% in cases of catheter shift, and lowest (14.3%) in cases of omental wrap.

### Risk factors for catheter failure

Compared with patients with catheter patency, those with catheter failure were significantly younger (37.9 ± 15.0 years vs. 47.9 ± 15.8 years), had lower BMI (20.7 ± 3.1 kg/m^2^ vs. 21.6 ± 3.2 kg/m^2^), and lower serum creatinine (9.7 [IQR:7.7–12.1] mg/dL vs. 10.7 [IQR: 8.4–13.7] mg/dL). Patients catheterized with coiled catheters had a significantly higher incidence of catheter failure than those with straight catheters (14.7% vs 3.4%, *P* = 0.014; Table 1). In the univariate Cox regression model, baseline younger age, lower BMI, and higher serum creatinine were risk factors for catheter failure. After adjustment for confounders, the multivariate Cox model shown that every 5 years increase in age was associated with 19% decrease of risk (hazard ratio [HR]: 0.81, 95%CI: 0.73–0.89) for catheter failure. Similar associations between these risk factors and functional catheter problems were also observed (Table 2). An additional analysis found that a significantly higher percentage of patients aged ≤50 years had functional catheter malfunctions caused by omentum wrap than did patients aged > 50 years (21.7% vs. 7.3%, *P* = 0.039).

**Table 2:** Risk factors for functional catheter problem and catheter failure in COX models

Variables	Univariate model		Multivariate model	
	HR (95%CI)	*P* value	HR (95%CI)	*P* value
Functional catheter problem				
Age (every 5 years increase)	0.86 (0.81–0.91)	< 0.001	0.86 (0.81–0.92)	< 0.001
Sex (Male)	1.08 (0.79–1.49)	0.623	1.01 (0.72–1.44)	0.942
BMI (kg/m^2^)	0.94 (0.89–0.99)	0.024	0.97 (0.92–1.03)	0.266
Serum creatinine (mg/dL)	1.04 (1.00–1.08)	0.023	1.03 (0.99–1.06)	0.934
Hemoglobin (g/dL)	0.92 (0.84–1.01)	0.068	0.96 (0.88–1.03)	0.346
BSA (m^2^)	0.66 (0.25–1.77)	0.410	–	–
Diabetic nephropathy	0.83 (0.55–1.24)	0.359	–	–
History of abdominal surgery	1.38 (0.81–2.35)	0.235	–	–
eGFR (ml/min/1.73m^2^)	0.96 (0.89–1.03)	0.227	–	–
Serum albumin (g/dL)	0.89 (0.65–1.23)	0.482	–	–
Catheter failure				
Age (every 5 years increase)	0.80 (0.74–0.87)	< 0.001	0.81 (0.73–0.89)	< 0.001
Sex (Male)	1.06 (0.67–1.68)	0.817	1.25 (0.75–2.10)	0.393
BMI (kg/m^2^)	0.91 (0.85–0.98)	0.017	0.94 (0.86–1.03)	0.164
Serum creatinine (mg/dL)	1.07 (1.02–1.12)	0.008	1.05 (0.98–1.10)	0.765
Hemoglobin (g/dL)	0.90 (0.79–1.03)	0.133	0.92 (0.81–1.04)	0.364
BSA (m^2^)	0.42 (0.10–1.74)	0.234	–	–
Diabetic nephropathy	0.83 (0.46–1.48)	0.517	–	–
History of abdominal surgery	1.55 (0.75–3.23)	0.241	–	–
eGFR (ml/min/1.73m^2^)	0.96 (0.87–1.07)	0.468	–	–
Serum albumin (g/dL)	0.97 (0.61–1.54)	0.886	–	–

Abbreviations: *BMI* body mass index, *BSA* body surface area, *eGFR* estimated glomerular filtration, *HR* hazard ratio, *CI* confidence interval

### Prevalence of other urgent-start PD related complications

For perioperative complications, significant hemorrhage presented in 3 (0.1%) patients and none suffered bowel perforation. Early peritonitis occurred in 24 patients (0.28 per patient-year), and early exit-site infections occurred in 7 patients (0.08 per patient-year). Details of the relevant organisms and outcomes of these episodes are provided in the additional file **(**Additional file 1: Table S1 and Table S2). For the 147 patients (7.1%) who experienced abdominal wall complications (including hernia, hydrothorax, hydrocele, pericatheter leakage, and subcutaneous leakage), 36 (24.5%) of these events happened within the first month after catheter insertion. Among the early-PD abdominal wall complications, pericatheter leakage was the most common complication, which responded well to conventional treatments; none resulted in technique failure. By contrast, hernia, hydrothorax, and hydrocele were the most common major complications of late-stage PD. Patients with hydrothorax were transferred to HD **(**Table 3**)**. After adjusting for confounders in multivariate analyses, male sex (HR: 1.43, 95%CI: 1.00–2.04), diabetic nephropathy (HR: 1.56, 95%CI: 1.08–2.25), and lower hemoglobin levels (HR:0.89, 95%CI: 0.81–0.98) were independent risk factors for abdominal wall complications (Table 4).

**Table 3:** Other complications related to urgent-start PD and outcomes in the entire cohort (*n* = 2059)

Complications	Overall (n)	Early present (n)^c^	Surgical Repair(n)^d^	Result in technique failure (n)^d^
Perioperative complications				
Bowel perforation	0	0	0	0
Significant hemorrhage^a^	3 (0.1%)	3 (0.1%)	2	0
Early infections^b^	31 (1.5%)	–	–	–
Peritonitis	24 (1.2%)	–	–	–
Exit-site infections	7 (0.3%)	–	–	–
Abdominal wall complications				
Hernia	74 (3.6%)	4 (0.2%)	18 (24.3%)	8 (10.8%)
Inguinal hernia	34 (1.7%)	3 (0.1%)	15 (44.1%)	5 (14.7%)
Umbilical hernia	40 (1.9%)	1 (0.05%)	3 (7.5%)	3 (7.5%)
Hydrothorax	18 (0.9%)	3 (0.1%)	0	18 (100%)
Hydrocele	29 (1.4%)	8 (0.4%)	4 (13.8%)	9 (31.0%)
Pericatheter leakage	19 (0.9%)	19 (0.9%)	0	0
Subcutaneous leakage	7 (0.3%)	2 (0.1%)	0	4 (57.1%)

Note. ^a^Hemorrhage requiring transfusion or surgical intervention;

^b^ Peritonitis and exit-site infections within 2 weeks after catheter insertion;

^c^ Present within the first month;

^d^ The percentage in each subtype of complications

**Table 4:** Risk factors for abdominal wall complications in COX models

Variables	Univariate model		Multivariate model	
	HR (95%CI)	*P* value	HR (95%CI)	*P* value
Age (every 5 years increase)	0.99 (0.95–1.06)	0.985	1.00 (0.95–1.06)	0.944
Sex (Male)	1.39 (0.98–1.97)	0.069	1.43 (1.00–2.04)	0.048
Diabetic nephropathy	1.59 (1.10–2.30)	0.013	1.56 (1.08–2.25)	0.017
History of abdominal surgery	0.51 (0.21–1.24)	0.136	0.50 (0.21–1.23)	0.133
Hemoglobin (g/dL)	0.90 (0.82–0.99)	0.027	0.89 (0.81–0.98)	0.016
BMI (kg/m^2^)	1.02 (0.97–1.08)	0.436	–	–
Type of catheter (coiled VS. straingt)	1.03 (0.56–1.92)	0.916	–	–
Serum creatinine (mg/dL)	1.01 (0.97–1.05)	0.665	–	–
BSA (m^2^)	1.71 (0.60–4.89)	0.315	–	–
eGFR (ml/min/1.73m^2^)	1.05 (0.94–1.08)	0.891	–	–
Serum albumin (g/dL)	0.86 (0.61–1.21)	0.379	–	–

Abbreviations: *BMI* body mass index, *BSA* body surface area, *eGFR* estimated glomerular filtration rate, *HR* hazard ratio, *CI* confidence interval

### Overall technique survival

**Fig. 5:**
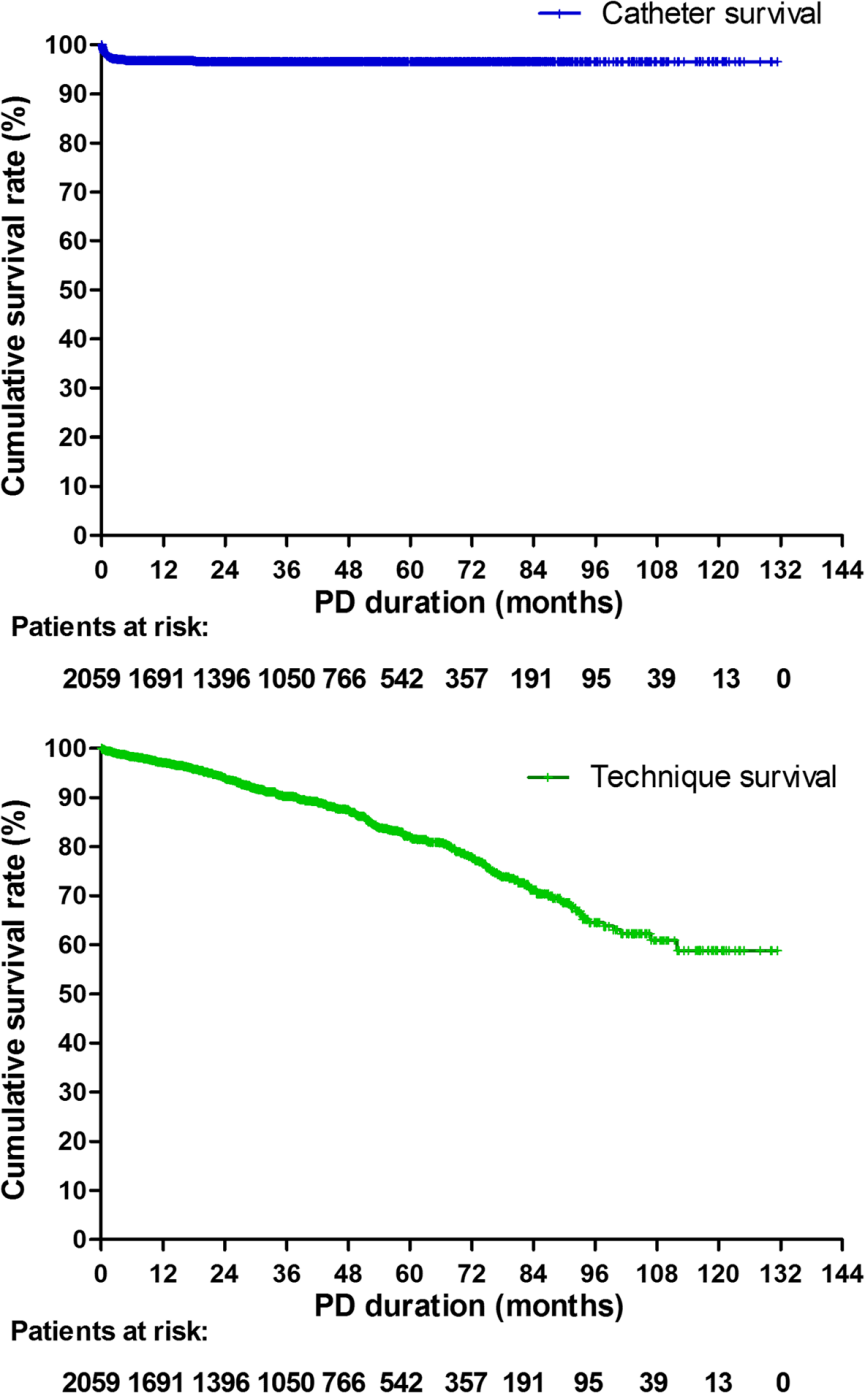
Catheter survival and death-censoring technique survival estimated by Kaplan-Meier analysis

During the follow-up period, 291 patients (14.1%) were transferred to HD, 430 (20.9%) received renal transplants, 534 (25.9%) died, and 738 (35.8%) remained on PD. The crude death-censoring technique failure rate was 0.04 per patient-year. Kaplan-Meier analysis showed that at the end of 1-month, 1-year, 3-year, 5-year, and 10-year, technique survival rate was 99.5, 97.0, 90.3, 82.7, and 58.8%, respectively (Fig. 5).

## Discussion

In the present cohort study of 2059 ESRD patients received urgent-start PD, their short-term and long-term rates for catheter failure and other catheter-related complications (such as bowel perforation, significant hemorrhage, early infections, and abdominal wall complications) were very low. Catheter patency rates were 1-month: 97.6%, 1-year: 96.4%, 3-year: 96.2% and 5-year: 96.2%; while technique survival rates were 1-month: 99.5%, 1-year: 97.0%, 3-year: 90.3%, and 5-year: 82.7%.

Catheter patency has been emphasized as the primary marker of successful PD catheter insertion; and ISPD guidelines recommend that the rate should be > 80% at 1 year [9]. Low catheter patency is a major concern in urgent-start PD because earlier-start PD may irritate the omentum and cause omental wrapping, thus leading to the catheter failure [9, 11, 21]. However, catheter patency rates were rarely reported in previous studies on urgent-start PD, whereas functional catheter problems (including catheter malfunction, catheter shift, and/or omental wrapping) reportedly fluctuated between 2.0 and 15.4% in previous studies [5, 11, 13, 14, 16]. In our study, 156 (7.6%) patients experienced functional catheter problems; 40.4% of their functional catheter problems occurred within 14 days after insertion, and 51.9% were rescued by the conservative methods. Only 3.6% of the catheters in our study cohort failed and needed surgical interventions. The 1-year catheter patency rate and technique survival rate in our study population was far in excess of the recommended target [9, 22]. By our experience, careful feedback and communication among the nephrologists, the PD nurses and the patients was helpful for promptly catching and solving functional catheter problems. We have a team with excellent nephrologists and nurses who responsible for the process of PD therapy from inpatient to outpatient treatment. Generally, catheter-related complications would be estimated by the PD nurses firstly and then diagnosed by nephrologists. The team would discuss whether the complications were related to catheter insertion technique, or insufficient training, or other causes. Finally, some potentially useful suggestions to solve the problems would be proposed. In addition, early initiation of low-volume PD may also help prevent catheter obstructions, and local adhesions which could eventually lead to catheter failure [23]. We also found that younger age was a risk factor independently associated with higher catheter failure in the current study. Further result showed that younger patients had higher percentages of omentum wrapping as a cause of functional catheter malfunctions. Perhaps younger patients have more active omentum, which may lead to a higher rate of omentum wrapping and subsequent catheter failure.

Abdominal wall complications (especially pericatheter leakage, which happens early after catheter insertion) are also major concerns in urgent-start PD [9]. Reported incidence of dialysate leakage varies from 1.5 to 37% in regular PD patients [5, 24–27], but fluctuates between 0 and 13.5% in patients who receive urgent-start PD [5, 11, 13–16]. In theory, increased intraperitoneal infusion during PD will increase the intraperitoneal pressure, and eventually increase the risk of developing abdominal wall complications. Our results shown that early pericatheter leakage only occurred in 19 (0.9%) patients in our cohort, and all these cases responded well to conservative treatments; none of them resulted in technique failure. As introduced in the present study, a double purse-string suture on the posterior rectus sheath and parietal peritoneum (Fig. 2) was used to fix the catheter and strengthen the “artificial weakness” at the catheter insertion points. In addition, a gradually increasing IPD regimen (Fig. 1) to start PD was used in the current study to prevent suddenly increased intraperitoneal pressure. The lower intra-abdominal pressure could prevent dialysate leaks and allow abdominal incisions time to heal after catheter insertion. In this way, the incidence of pericatheter leakage was successfully decreased. These results suggest that urgent-start peritoneal dialysis can be effectively conducted with an acceptable rate of abdominal wall complications. Our study also identified that male sex, diabetic nephropathy and lower hemoglobin levels were independent risk factors for abdominal wall complications, which indicates that we should pay more attention to these patients at risk for assessment and management before initiating PD, and during follow-up period.

Early infection is another concern about urgent-start peritoneal dialysis. A Danish study reported that whereas 15.4% of urgent-start peritoneal dialysis patients suffered peritonitis within the first 3 months, 15.4% of the planned PD group also suffered peritonitis [11]. However, in the present study, early peritonitis occurred only in 1.2% of our patients, and the long-term overall incidence of peritonitis was 0.16 cases per patient-year, as reported in our previous study [28]. In our center, a single-dose of prophylactic antibiotic was given 30 min before catheterization. All incident PD patients and their caregivers would undergo standard PD training. Taken together, strict sterilization procedure, preoperative antibiotic prophylaxis, experienced nephrologists for catheter insertion, and a professional nursing team to train patients and caregivers may help control occurrence of early peritonitis.

Several limitations should be taken into consideration in this study. First, selection bias was inevitable for a single-center cohort study. Second, as a retrospective study, we could not control some potential confounders, such as variations among catheter insertion techniques by different operators. Third, as we lacked a control group, our study still cannot show whether urgent-start peritoneal dialysis is better or inferior to planned PD for patients who can wait for planned PD without receiving HD.

## Conclusion

Our results over 10 years show that ESRD patients who received urgent-start peritoneal dialysis had high rates of catheter patency and technique survival, and low incidences of catheter-related complications, which indicates that urgent-start peritoneal dialysis is safe, efficacious, and practical for patients with ESRD. A unique infrastructure and management approach that includes a well-trained PD team, a standardized procedure for catheter insertion by experienced nephrologists, a carefully designed initial PD prescription, a well-planned training curriculum for patients and their caregivers, and comprehensive follow-up care appear to be essential for the successful urgent-start peritoneal dialysis program. Clinical outcomes of unplanned versus planned dialysis starters among different PD centers warrant prospective study.

## Additional files

Additional file 1: **Table S1.** Details of the organisms and the outcomes of the eraly peritonitis. **Table S2.** Details of the organisms and the outcomes of the early exit-site infections. (DOCX 13 kb)

## Data Availability

The datasets used and/or analyzed during the current study are available from the corresponding author on reasonable request.

## Abbreviations

BMI: Body mass index
BSA: Body surface area
CAPD: Continuous ambulatory peritoneal dialysis
CKD: Chronic kidney disease
ESRD: End stage renal disease
HD: Hemodialysis
HR: Hazard ratio
IPD: Intermittent peritoneal dialysis
IQR: Interquartile range
PD: Peritoneal dialysis
SD: Standard deviation
USRDS: United States Renal Data System
