# Supplementary material for: Urgent-start peritoneal dialysis for patients with end stage renal disease: a 10-year retrospective study
Source: BMC Nephrol. 2019 Jul 2;20:238. doi: 10.1186/s12882-019-1408-9 (PMC6604308; doi:10.1186/s12882-019-1408-9)
Supplement: Supplementary file 1 — Table S1. Details of the organisms and the outcomes of the eraly peritonitis. Table S2. Details of the organisms and the outcomes of the early exit-site infections. (DOCX 13 kb) [file 12882_2019_1408_MOESM1_ESM.docx]

**Additional file 1**

Additional file 1: Table S1 Details of the organisms and the outcomes of the eraly peritonitis (n=24)

| Organisms | Numbers | Outcomes |
| --- | --- | --- |
| Klebsiella pneumoniae | 1 | resolved |
| Streptococcus | 1 | resolved |
| Enterobacter cloacae | 1 | resolved |
| Pseudomonas Schwann | 1 | resolved |
| Staphylococcus haemolyticus | 1 | resolved |
| Staphylococcus epidermidis | 1 | resolved |
| Staphylococcus capitulum | 1 | resolved |
| Acinetobacter Bauman | 1 | resolved |
| Escherichia coli | 4 | resolved |
| Culture negative | 12 | resolved |

Additional file 1: Table S2 Details of the organisms and the outcomes of the early exit-site infections (n=7)

| Organisms | Numbers | Outcomes |
| --- | --- | --- |
| Staphylococcus aureus | 3 | resolved |
| Culture negative | 3 | resolved |
| Not done | 1 | resolved |
